# Maternal substance use and integrated treatment programs for women with substance abuse issues and their children: a meta-analysis

**DOI:** 10.1186/1747-597X-5-21

**Published:** 2010-09-01

**Authors:** Karen Milligan, Alison Niccols, Wendy Sword, Lehana Thabane, Joanna Henderson, Ainsley Smith, Jennifer Liu

**Affiliations:** 1 Integra, (25 Imperial Street.), Toronto, ON, (M5P 1B9), Canada; 2 Department of Psychiatry and Behavioural Neurosciences, McMaster University, McMaster Children's Hospital - Chedoke site, (565 Sanatorium Road.), Hamilton ON, (L8N 3Z5), Canada; 3 School of Nursing, McMaster University (1200 Main Street West), Hamilton ON, (L8N 3Z5), Canada; 4 Department of Clinical Epidemiology and Biostatistics, McMaster University, St. Joseph's Healthcare, (50 Charleton Avenue East), Hamilton ON, (L8N 4A6), Canada; 5 Centre for Addiction and Mental Health, University of Toronto, (33 Russell Street), Toronto, ON, (M5 S 2S1), Canada; 6 Department of Psychiatry and Behavioral Neurosciences, McMaster University, McMaster Children's Hospital - Chedoke site (565 Sanatorium Road), Hamilton, ON, (L8N 3Z5), Canada; 7 Department of Mathematics and Statistics, McMaster University (1200 Main Street West), Hamilton ON, (L8N 3Z5), Canada

## Abstract

**Background:**

The rate of women with substance abuse issues is increasing. Women present with a unique constellation of risk factors and presenting needs, which may include specific needs in their role as mothers. Numerous integrated programs (those with substance use treatment and pregnancy, parenting, or child services) have been developed to specifically meet the needs of pregnant and parenting women with substance abuse issues. This synthesis and meta-analysis reviews research in this important and growing area of treatment.

**Methods:**

We searched PsycINFO, MedLine, PubMed, Web of Science, EMBASE, Proquest Dissertations, Sociological Abstracts, and CINAHL and compiled a database of 21 studies (2 randomized trials, 9 quasi-experimental studies, 10 cohort studies) of integrated programs published between 1990 and 2007 with outcome data on maternal substance use. Data were summarized and where possible, meta-analyses were performed, using standardized mean differences (*d*) effect size estimates.

**Results:**

In the two studies comparing integrated programs to no treatment, effect sizes for urine toxicology and percent using substances significantly favored integrated programs and ranged from 0.18 to 1.41. Studies examining changes in maternal substance use from beginning to end of treatment were statistically significant and medium sized. More specifically, in the five studies measuring severity of drug and alcohol use, the average effect sizes were 0.64 and 0.40, respectively. In the four cohort studies of days of use, the average effect size was 0.52. Of studies comparing integrated to non-integrated programs, four studies assessed urine toxicology and two assessed self-reported abstinence. Overall effect sizes for each measure were not statistically significant (*d *= -0.09 and 0.22, respectively).

**Conclusions:**

Findings suggest that integrated programs are effective in reducing maternal substance use. However, integrated programs were not significantly more effective than non-integrated programs. Policy implications are discussed with specific attention to the need for funding of high quality randomized control trials and improved reporting practices.

## Background

Rates of substance abuse in women are on the rise [1-4]. Research suggests that women are more vulnerable to the adverse physiological consequences associated with substance abuse [5]. Substance abuse in women is also associated with a unique constellation of risk factors and needs, including increased prevalence of mental health problems, histories of physical or sexual abuse [6,7], serious medical problems, poor nutrition, relationship problems (including domestic violence), and deficits in social support [8,9]. These unique risk factors and presenting needs of women have resulted in the development of numerous women-specific comprehensive treatment models that address the full range of needs and include components such as trauma-specific and trauma informed therapy [4].

In addition to adjusting our lens to sharpen our focus on the unique needs of women, there is also a need to understand women who abuse substances in their role as mothers. The majority of women who abuse substances are of child- bearing age [10]. As such, substance abuse also has implications for child health and parenting. Children born to women who used substances during pregnancy are at greater risk for prematurity, low birth weight, impaired physical growth and development, behavioral problems, learning disabilities, and substance use [2,11]. Women who continue to abuse substances after childbirth, despite their best intentions are at risk for a wide range of parenting deficits [12].

Given the specific risks and needs of women with substance abuse issues and their children, researchers, clinicians, and policy makers have recommended that substance use treatment programs address women's physical, social, and mental health needs, as well as children's needs through prenatal services, parenting programs, child care, and other child-centered services [13-15]. This recognition has resulted in the development of numerous integrated (or comprehensive) treatment programs (those that include on-site pregnancy-, parenting-, or child-related services with addiction services) in countries, such as the United States and Canada.

A theoretical rationale for including pregnancy-, parenting-, or child-related services with substance use services is that integrated treatment programs may enhance the impact of substance use treatment because a) integrated programs may reduce barriers to engaging and remaining in treatment (such as lack of adequate child care [16]), b) integrated interventions may have a synergistic effect (e.g., mental health services for mother may improve mood which may be associated with reduced substance use), and c) parenting and child development services may increase maternal motivation to reduce substance use. Certainly in their development and evaluation of integrated programs, The Centre for Substance Abuse Treatment [17] has suggested that "treatment that addresses the full range of a woman's needs is associated with increased abstinence and improvement in other measures of recovery, including parenting skills and overall emotional health. Treatment that addresses alcohol and other drug abuse only may well fail and contribute to a higher potential for relapse."

As the number of integrated programs has grown over the past 20 years, empirical evidence about the effectiveness of these programs has accumulated. Although some individual studies examining the effectiveness of integrated treatment programs suggest positive outcomes, the study quality varies, ranging from randomized controlled trials to less rigorous single-group designs. As such, questions remain regarding the robustness of treatment effects relative to non-integrated substance use programs. Many studies have been limited by inadequate statistical power (small sample size), complicating interpretation of results.

A few systematic reviews and a meta-analysis examining outcomes associated with gender specific (women-only) treatment programs have been completed. In a systematic review of 38 studies on substance abuse treatment for women, Ashley et al. [2] examined six specific components of treatment programs. Programs with prenatal care, child care, and parenting were associated with higher rates of abstinence and reduced substance use. Orwin, Francisco, and Bernichol [18] conducted a meta-analysis of studies on the effects of substance abuse treatment for women on substance use, maternal well-being, and pregnancy outcomes. Findings suggested that enhancing women-only treatment programs with prenatal care or therapeutic child care added value above and beyond the effects of standard women-only programs. However, neither of these studies specifically focused on integrated programs and they did not include the recent proliferation of studies of integrated programs.

Synthesizing current research on women-specific programs that include child and/or parenting components (i.e., integrated programs) is a pressing task given that 1) increased funding is being directed towards supporting integrated treatment programs, 2) a proliferation of programs have been developed, and 3) an increased number of evaluations have been conducted. Before more resources are spent on these programs and research, existing literature needs to be synthesized to enhance our knowledge and delineate priorities and directions for future research (cf. Cooper & Hedges [19]). While a synthesis does not provide a conclusive statement about a problem or treatment area, it can provide pivotal information for the field on what can be improved. Precise and reliable research syntheses will assist in ensuring that the next wave of primary research is sent off in the most illuminating direction [19].

Meta-analysis is well suited to the task of research synthesis and to addressing the limitations in the current literature. First, meta-analysis addresses the problem of low statistical power by allowing the results of small-sample studies to be combined, resulting in increased statistical power. Dennis, Huebner, and McLellan [20] found that 87% of the studies in Edwards and Steinglass's [21] meta-analysis of alcoholism interventions did not meet the minimum level of acceptable power, thus placing them at high risk for missing existing treatment effects.

Similarly, in our evaluation of New Choices, an integrated outpatient program, many results were moderate in strength but failed to reach statistical significance [22]. Thus, meta-analysis can increase interpretability of findings and allow more reliable conclusions about treatment effectiveness. Second, the strength of the intervention effect can be determined by meta-analysis through the use of effect size statistics. The strength of observed effects is less influenced by statistical power than tests of significance and is more clinically relevant [23]. Third, the generalizability of findings from a meta-analysis is greater than that of findings from individual studies because meta-analytic findings are based on a diverse set of study samples rather than a single study sample [24]. Fourth, unlike qualitative reviews, meta-analysis allows one to statistically determine if the strength of the treatment effects differs significantly among studies and then to quantitatively examine what factors, such as program, client, and study characteristics, may be responsible for these differences. For example, variations in study quality can be examined statistically for their potential impact on study findings.

Meta-analysis is an appropriate way to combine results even in circumstances where there are few studies and, in fact, the situation is not uncommon. A common misconception is that meta-analysis is applicable only to research areas involving large numbers of studies. However, meta-analysis can be applied effectively to a small number of studies on a focused topic [25]. Some have argued that focused meta-analyses are more relevant to informing policy [26] and several meta-analyses in the field of substance abuse treatment reflect this approach [25]. According to Cooper and Hedges [19],

If the research question is important, it would be interesting to know how much research there is on the problem, even if the answer was none at all... Ultimately the arbiter of whether a synthesis is needed will not be numerical standards, but the fresh insights a synthesis can bring to a field. Indeed, although a meta-analysis cannot be performed without data, many social scientists see value in empty syntheses that point to important gaps in our knowledge.

In this paper, we examine the impact of integrated treatment programs on maternal substance use. We hypothesize that participation in integrated programs is associated with significant improvements in maternal substance use outcomes and that maternal substance use outcomes are significantly better for women participating in integrated programs than women participating in non-integrated programs. We examine the strength of these effects and, if there is variability in effects among studies, we examine client, program, and study characteristics that may moderate the impact of treatment.

## Method

### Literature search

We used three main strategies to identify outcome studies of intervention programs for women with substance use issues and their children: online bibliographic database searches; checking printed sources; and requests to researchers (cf., Mullen [27]; Rosenthal [23]). First, we searched relevant bibliographic databases (PsycINFO, MedLine, PubMed, Web of Science, EMBASE, Proquest Dissertations, Sociological Abstracts, and CINAHL) for studies published in English, using the terms "substance use/abuse," "addiction," "alcoholism," "intervention," "treatment,", "therapeutic," "rehabilitation," "women," "child," "mother," "infant," "mental health," "parenting," and "prenatal" (singly and in combination).

Secondly, we examined reference lists of retrieved articles for potentially relevant documents. In addition, we manually searched relevant journals in the area (Journal of Substance Abuse Treatment, Journal of Substance Use, Substance Use and Misuse, Journal of Psychoactive Drugs, Addiction, Journal of Drug Issues, The International Journal of the Addictions, Addictive Behaviors, and the Journal of Substance Abuse). Documents that appeared to be relevant on the basis of titles or abstracts were retrieved.

Finally, we searched for grey data (technical reports, unpublished data) to ensure our review was not biased to published sources. All researchers identified through all search strategies described, as well as researchers presenting at relevant conferences identified using Google and Cross Currents (Upcoming Events), were contacted by email to request any relevant published or unpublished data. Of the 200 researchers identified and emailed, 48% responded and 28 additional studies were identified. In total, 327 studies were retrieved and coded for eligibility.

### Eligibility criteria and study inclusion

**Figure 1:**
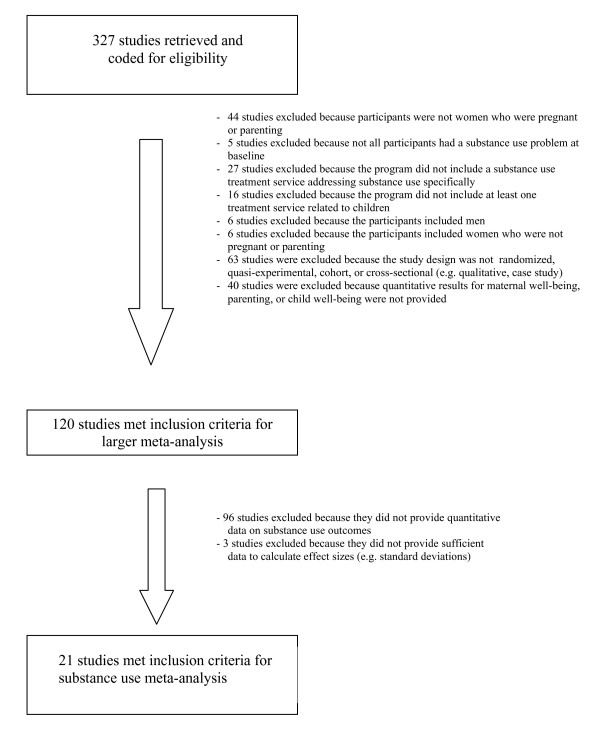
**Study Eligibility Flowchart**.

Figure 1 depicts the process and outcomes of eligibility coding. Studies were included in our larger meta-analysis project if:

1) study participants were women who were pregnant or parenting;

2) all study participants had substance use problems at baseline;

3) the treatment program included at least one substance use treatment and at least one child (< 16 years) treatment service (e.g., prenatal care, child care, parenting classes);

4) the program was not for men or for women not pregnant or parenting;

5) the program was not a smoking cessation program; and

6) quantitative data were provided on length of stay, treatment completion, maternal substance use, maternal well-being, or child well-being.

Using these criteria, 120 studies were considered eligible for inclusion in the meta-analysis. Based on a random sample of 20% of the studies, inter-rater reliability for eligibility coding was high, *Kappa *= 0.81. Discrepancies were resolved by consensus.

The completeness of the search was estimated using the capture re-capture method [28-30]. Based on this method, the estimated number of missing articles is 8 (95% CI: 2, 24), which suggests a 90% capture rate (i.e., the identified studies cover 90% of the horizon). This reasonably high capture rate suggests that a sufficient number of studies were retrieved to avoid bias in the results of the meta-analysis.

Few of the studies involved comparison groups. Of the 120 studies, 12 were randomized trials (5 comparing integrated to non-integrated programs) and 25 were quasi-experimental studies (9 comparing integrated to non-integrated programs).

This paper focuses on those studies that examined changes in maternal substance use as such only randomized trials and quasi-experimental studies comparing integrated programs to non-integrated programs or no treatment control groups and cohort studies (i.e., pre-post studies) that reported data on maternal substance use outcomes were included in the present systematic review and analyses. As detailed below, there were 3 randomized trials (*n *= 250), 9 quasi-experimental studies (*n *= 2105), and 10 cohort studies *(n *= 856).

### Coding procedures

We developed a codebook for this systematic review and meta-analysis based on theoretical models of treatment, literature review, and data availability. The codebook was pilot tested by project staff and investigators and revised during early coding. Variables were added or deleted, and decisions and clarification of specific variables were recorded in a coding policy manual.

We coded study context (author, document date, type of document, country), methodology (sample size, attrition, study design), participant characteristics (age, marital status, education, employment, income, substance abuse history, previous substance abuse treatment, mental and physical health, involvement with the legal system), child characteristics (age, custody, involvement with child protection services, positive toxicology at birth), treatment program characteristics (population served, planned length of treatment, intensity of treatment, location, services), dependent variable characteristics (type of outcome measure, type of data), and effect size calculation statistics. There were considerable missing data (especially on client characteristics and program services) and limited quantitative data on outcomes (e.g., standard deviations, sample sizes). In an attempt to obtain missing data, we contacted four researchers, three of whom responded, with two providing additional data.

Each study was coded by a trained research assistant (AS), who met frequently with the principal investigator (KM) during the development of the codebook and early stage of coding. AS coded all studies and 20% of studies reporting on maternal substance use outcomes were coded by both AS and KM. Kappa and percent agreement were calculated for all variables. There was 100% agreement for identification of dependent variables. For program and participant variables, there was 94% mean agreement for continuous variables and a *Kappa *of. 0.97 for categorical variables. Discrepancies were resolved by consensus.

### Study quality

The Jadad Scale [31,32], widely used in the medical literature, was used to assess the quality of randomized trials. On the Jadad Scale, studies are rated on a scale from 0 to 5, with the highest possible score (5) given for those with descriptions of: 1) randomization; 2) an appropriate method of randomization; 3) double-blinding; 4) an appropriate method of double-blinding; and 5) withdrawal and dropouts.

The Newcastle-Ottawa Scale (NOS [33]) was used to assess the quality of non-randomized studies. On the NOS, studies are rated on a scale from 0 to 9 on the basis of three main issues: study group selection; group comparability; and outcome ascertainment. The content validity and inter-rater reliability for the NOS have been established and further evaluation is being conducted [33].

A trained research assistant (AS) and Master's student (JL) coded study quality under the supervision of co-authors AN and LT. Inter-rater reliability (based on 20% of the included studies) was high, *Kappa *= 0.80. Discrepancies were resolved by consensus.

### Calculating and combining effect sizes

We transformed results from each study to the standardized mean difference (Cohen's *d*) using Comprehensive Meta-analysis II [34]. The standardized mean difference is computed by subtracting the mean outcome score of the comparison group from that of the treatment group and dividing the difference by the pooled standard deviation. The effect size calculation was based on the number of participants in the analysis (corrected for attrition). By convention, an outcome for which the (integrated) treatment group showed more improvement than the comparison group was indicated by a positive sign, whereas an outcome that favored the comparison group was indicated by a negative sign. Effect sizes were corrected by inverse variance weights based on standard error.

When combining effect sizes, we computed both fixed and random effects to calculate estimates of the impact of treatment on outcomes across studies [35]. Chi-square tests of homogeneity were used to assess if results significantly differed among studies. When significant heterogeneity was found, random effects findings rather than fixed effects findings were used [36].

File drawer statistics, which represent the number of unretrieved studies averaging null results (i.e., not supporting the pattern established by research findings) that would be required to reduce the significance of the meta-analytic finding to the just significant level, *alpha *= .05 [23], were calculated to assess publication bias.

### Moderator analyses

Where there was significant heterogeneity among studies, we explored factors that may have moderated the effect of treatment on outcomes using analyses of variance or regression analyses, depending on the type of moderating variable (categorical or continuous). Potential moderators included client characteristics (e.g., maternal age, education, socioeconomic status, number of children, age of children, length of stay in treatment), program characteristics (e.g., residential or not, types of program services provided, targeted substance, whether or not children reside), and study characteristics (e.g., design, quality), as have been examined in previous studies [37,38].

## Results

### Descriptive information

There was variability across studies in terms of study design and substance use measures. In terms of study design, there were 3 randomized trials (*n *= 250;[39-41]), 9 quasi-experimental studies (*n *= 2105; [42-50]), and 9 cohort studies *(n *= 856, [22,51-57] Kerwin, treatment outcome data for women in substance abuse group, Unpublished data]). One quasi-experimental study [56] compared two integrated treatments. Given our research questions, these groups were examined separately and included in the cohort data. Substance use measures included urine toxicology and self report measures, (e.g., Addiction Severity Index), percent of participants abstinent from substance use, frequency of use, cost of addiction, negative outcomes of addiction, and change in use. Given this heterogeneity, we present effect size information for all studies (see Tables 1, 2 and 3) and only combine effect sizes for meaningfully similar measures of maternal substance use.

**Table 1:** Studies comparing integrated treatment to no treatment

Study	n	Groups	Design	Measure	*d (SE)*	*p*	Study Quality
Armstrong & Osejo^43^	Treatment: 782 Control: 610	Integrated outpatient treatment vs no treatment	Quasi- Experimental	% positive urine toxicology	0.18 (0.07)	0.007	5/9
Whiteside-Mansell & Crone^44^	Treatment: 72 Control: 23	Integrated Residential treatment vs no treatment	Quasi- Experimental	% using alcohol	0.49 (0.22)	0.022	2/5
				% using drugs	1.41 (0.42)	0.001	

Significance of d based on test of null (z-test).

**Table 2:** Cohort studies examining changes in maternal substance use

Study	n	Measure	*d (SE)*	*P*	Study Quality
Conners & Bradley^60^	62	Number of days of drug use	0.74^† ^(0.15)	0.001	2/9
	62	% using substances	2.90 (0.80)	0.001	
Elk & Schmitz^61^	19	Addiction Severity Index Alcohol Composite	0.48^‡ ^(0.24)	0.049	0/9
	19	Addiction Severity Index Drug Composite	0.83* (0.27)	0.002	
Evenson & Binner^62^	98	CSTAR Alcohol and Drug Problem Inventory	0.72 (0.11)	0.001	0/9
	98	Cost of Alcohol	0.58 (0.11)	0.001	
	98	Cost of Drugs	0.74 (0.11)	0.001	
	98	CSTAR Substance Use Questionnaire - Did not use substances	2.01 (0.23)	0.001	
	98	CSTAR Substance Use Questionnaire - Main alcohol/drug abstinent	1.60 (0.21)	0.001	
	98	CSTAR Substance Use Questionnaire - Alcohol Abstinent	1.72 (0.26)	0.001	
	98	CSTAR Substance Use Questionnaire - Alcohol Intox Abstinent	2.09 (0.35)	0.001	
	98	CSTAR Substance Use Questionnaire - Cannabis Abstinent	1.88 (0.26)	0.001	
	98	CSTAR Substance Use Questionnaire - Cocaine Abstinent	2.71 (0.79)	0.001	
Ingersoll & Knisely (low psychopathology group)^63^	33	Addiction Severity Index Alcohol Composite	0.29^‡ ^(0.18)	0.102	2/9
	33	Addiction Severity Index Drug Composite	0.92* (0.21)	0.000	
Ingersoll & Knisely (high psychopathology group)^63^	13	Addiction Severity Index Alcohol Composite	0.70 (0.31)	0.023	2/9
	13	Addiction Severity Index Drug Composite	0.28* (0.28)	0.322	
Kerwin (Treatment outcome data for women in substance abuse treatment group, Unpublished data)	7	Addiction Severity Index Alcohol Composite	0.51^‡ ^(0.32)	0.110	n/a
	7	Addiction Severity Index Drug Composite	0.56* (0.32)	0.087	
McClellan & Gutman^64^	529	Number of days of alcohol use	0.36^† ^(0.05)	0.001	2
	529	Addiction Severity Index Alcohol Composite (6 months follow up)	0.39 (0.05)	0.001	2
	529	Addiction Severity Index Alcohol Composite (12 months follow up)	0.39^‡ ^(0.05)	0.001	2
	529	Addiction Severity Index Drug Composite (6 month follow up)	0.63 (0.05)	0.001	2
	529	Addiction Severity Index Drug Composite (12 months follow up)	0.65* (0.05)	0.001	2
	529	Money spent on drugs (6 months follow up)	0.21 (0.04)	0.001	2
	529	Money spent on drugs (12 months follow up)	0.22 (0.44)	0.001	2
Niccols & Sword^22^	9	% using alcohol (3 months into program)	0.31 (0.48)	0.520	
	7	% using alcohol (6 months into program)	0.31 (0.52)	0.552	
	9	% using cannabis(3 months into program)	0.99 (0.53)	0.061	
	7	% using cannabis (6 months into program	0.99 (0.57)	0.083	
	9	% using cocaine (3 months into program)	1.17 (0.86)	0.175	
	7	% using cocaine (6 months into program	1.04 (0.87)	0.231	
	9	% using tranquilizers (3 months into program)	0.73 (0.90)	0.412	
	7	% using tranquilizers (6 months into program	0.60 (0.90)	0.502	
	9	% using crack (3 months into program)	0.73 (0.90)	0.412	
	7	% using crack (6 months into program	0.60 (0.90)	0.502	
	9	% using barbiturates (3 months into program)	0.73 (0.90)	0.412	
	7	% using barbiturates (6 months into program	0.60 (0.90)	0.502	
	9	% using over the counter drugs (3 months into program)	0.00 (0.53)	1.000	
	7	% using over the counter drugs (6 months into program	-0.65 (0.54)	0.239	
	9	% using other drugs (3 months into program)	0.51 (0.63)	0.419	
	7	% using other drugs (6 months into program	0.51 (0.69)	0.461	
Volpicelli & Markman^42^	21	Number of days of cocaine use	0.753^† ^(0.247)	0.002	1/9
	21	Number of days of cocaine use	0.423^† ^(0.228)	0.063	1/9
Wexler & Cuadrado^65^	44	% using alcohol	0.37 (0.28)	0.191	
	44	% using drugs	3.06 (0.81)	0.001	

^†^Study included in the overall effect size for number of days of substance use

^‡^Study included in the overall effect size for Addiction Severity Index Alcohol Composite

*Study included in the overall effect size for Addiction Severity Index Drug Composite

◊ Comprehensive Substance Abuse Treatment and Rehabilitation

Significance of d based on test of null (z-test).

**Table 3:** Studies comparing integrated to non-integrated treatment

Study	n	Groups	Design	Measure	*d (SE)*	*p*	Study Quality
Barkauskas and Low^46^	Treatment: 52 Control: 73	Residential program for pregnant, incarcerated women vs standard prison care for women	Quasi- Experimental	% positive toxicology screens	-0.29^†^ (0.69)	0.669	5/9
Carroll and Chang ^40^	Treatment: 7 Control: 7	Enhanced methadone treatment for women vs standard methadone treatment for women	Randomized Trial	% positive toxicology screens	-0.14^†^ (0.67)	0.83	1/5
Chang and Carroll ^47^	Treatment: 6 Control: 6	Enhanced methadone treatment for women vs standard methadone treatment for women	Quasi- experimental	% positive toxicology screens	0.43^†^ (0.70)	0.54	3/9
Gwadz and Leonard^39^	Treatment: 51 Control: 58	Integrated outpatient treatment for women vs standard outpatient treatment for women	Randomized Trial	National Alcohol Survey - Frequency of Alcohol Use	-0.12 (0.19)	0.545	1/5
				Drug Use Screen Inventory - Alcohol/Dru g Problems	0.077 (0.19)	0.69	
				Risk Behaviour Assessment - Frequency of Drug Use	-0.26 (0.19)	0.183	
Harshman^66^	Treatment: 25 Control: 27	Residential Integrated treatment for women vs Co-ed Residential Standard treatment	Quasi- experimental	# months since last used substances	-0.15 (0.28)	0.593	2/9
				SASSI-2 * Alcohol use	0.61 (0.28)	.033	
				SASSI-2 drug use	0.06 (0.28)	0.83	
Luthar et al. ^41^	Treatment: 60 Control: 67	Maternal psychotherapy plus standard methadone treatment vs. Recovery training plus standard methadone treatment	Randomized Trial	Positive toxicology screens - opiate use (follow up phase - up to one year follow up)	-0.08^† ^(0.18)	0.647	3/9
				Positive toxicology screens - cocaine use (treatment phase)	0.25 (0.18)	0.170	
				Positive toxicology screens - cocaine use (follow up phase - up to one year follow up)	-0.14 ^† ^(0.18)	0.428	
Sacks and Sacks ^67^	Treatment: 49 Control: 49	Residential integrated treatment for women vs co-ed standard residential treatment	Quasi- experimental	Substance Use Composite (incl. Drug use, frequency of use, # days of use)	0.12◊	NS	3/9
Sowers and Ellis^48^	Treatment: 26 Control: 15	Residential integrated treatment for women vs standard day treatment (not specified if women only)	Quasi- experimental	% not using any substances	0.33 (0.36)	0.36	4/9
Suchman and Mayes ^49^	treatment: 25 control: 23	Women only outpatient treatment with parenting intervention vs standard women only outpatient treatment	Quasi- Experimental	% not using any substances	0.15 (0.30)	0.628	3/9
Touissaint and VanDeMar k^68^	Treatment: 64 Control: 106	Residential Integrated Treatment for women vs Co-ed Residential Standard Treatment	Quasi- experimental	Addiction Severity Index Drug Composite	0.30 (0.16)	.059	6/9
				Addiction Severity Index Alcohol Composite	0.28 (0.16)	0.078	6/9

† Included in overall effect size for toxicology screens

* Substance Abuse Subtle Screening Inventory-2

◊ effect size reported, data on SE and *p *not available

Significance of d based on test of null (z-test)

### Study Quality

Study quality scores showed little variability among studies. Jadad Scale scores for the three randomized trials were 1, 2, and 3 (absolute range 0-5), which are considered poor to moderate scores. One study [40] was described as randomized but did not provide a description of randomization and was not double blind, as participants were aware of the treatment allocation. This study also did not provide a description of withdrawal and dropouts. The second study [39] was described as randomized but the method of randomization was not described. The study was not described as double blind but did provide a description of withdrawal and dropouts. The third study [41] was described as randomized and an appropriate method of randomization was used. This study was not described as being double blind but did provide a description of withdrawal and dropouts. NOS scores for the quasi-experimental studies varied from 2 to 6 (maximum possible score = 9), which are low to moderate scores. The Jadad Scale Score for the cohort study also was 1. The study [56] was described as randomized but did not provide a description of the method of randomization. The study was not described as being double blind and no description of drop-outs or withdrawal was provided. Newcastle-Ottawa Scale Scores for the cohort studies ranged from 0 to 5, which are low to moderate scores. It was unclear if these low scores were due to poor study quality or reporting (e.g., if there was no description of the ascertainment of treatment exposure, then this item was scored as 0).

### Studies of the impact of integrated programs on maternal substance use

Two studies compared substance use outcomes for women participating in integrated programs to women in no treatment control groups. In a quasi-experimental study, Armstrong et al. [42] examined percent negative urine screens in 782 integrated program clients and 610 no-treatment control participants and found that women participating in integrated programs were significantly more likely than women not in treatment to have negative urine toxicology screens during pregnancy *(d *= 0.18, *SE *= 0.07, *2.719 p *< .01). In a quasi-experimental study of 72 women in integrated programs and 23 women not in treatment, Whiteside-Mansell, Crone, and Conners [43] examined the percent using drugs and the percent using alcohol at the time of the birth of their child. Results indicated that significantly fewer women in integrated programs used drugs or alcohol than those not in treatment (*d *= 1.41 (*SE *= 0.42, *z *= *3.351, p *< .001 and *d *= 0.49, *SE *= 0.21, *z= *2.287, *p *= 0.02, for drug and alcohol use, respectively). See Table 1 for further study information.

**Figure 2:**
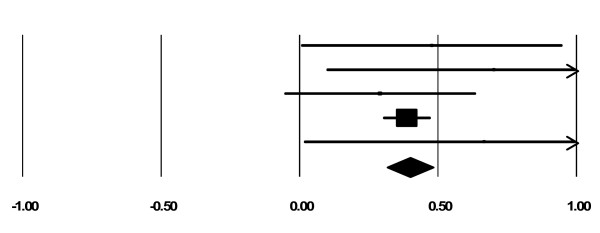
**Forest Plot for ASI Alcohol Composite**.

**Figure 3:**
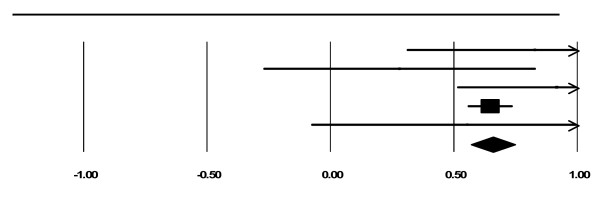
**Forest Plot for ASI Drug Composite**.

There were 10 cohort studies with data on maternal substance use at intake and end of treatment or follow up. As can be seen in Tables 2 and 3, 29 out of 31 measures indicated decreased maternal substance use. We combined studies with the most common measures of maternal substance use (i.e., Alcohol and Drug Composites of the Addiction Severity Index and days of use). Five studies involved the Alcohol and Drug Composites of the Addiction Severity Index, on which women in integrated programs reported significantly reducing their alcohol and drug use from intake to the end of treatment. The overall effect sizes using a fixed effects model were 0.40 (*z= *9.34, *p *< .001) for the alcohol composite and 0.65 (*z= *14.57, *p *< .001) for the drug composite (*CI*s = - 0.31 to 0.48 and 0.57 to 0.74, respectively). See Figures 2 and 3. These effect sizes are considered medium (Cohen, 1988). The file drawer statistic indicated that 66 and 143 studies, respectively, with null results would be required to reduce significance to the just - significant level, *alpha *= 0.05 (Rosenthal, 1991). This exceeds Rosenthal's critical value of 35 (5*k *+ 10, where *k *is the number of included studies). Therefore, we can be confident that these significant results would not be negated by null findings that were not included in the present analysis. Cochran's chi square test, which examines homogeneity of variance, was not statistically significant for alcohol (*Q *(4) = 1.58, *p *= 0.81 and drug (*Q *(4) *= *3.90, *p *= 0.42) composites.

**Figure 4:**
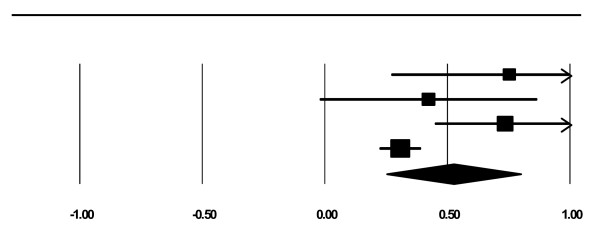
**Forest Plot for Days of Use**.

Four studies reported on days of use. Results indicated that women in integrated programs reported significantly reducing the number of days using substances from intake to the end of treatment, *z *= 3.74, *p *< .0001. The overall effect size using a random effects model was 0.52 (*CI *= 0.25 to 0.80), which is medium [58]. See Figure 4. The file drawer statistic indicated that 80 studies with null results would be required to reduce significance to just the significant level, *alpha *= 0.05 [23]. This exceeds Rosenthal's critical value of 30 (5*k *+ 10, where *k *is the number of included studies). Therefore, we can be confident that this significant result would not be negated by null findings that were not included in the present analysis. Given that Cochran's chi square test indicated significant heterogeneity between studies (*Q *(3) = 10.43, *p *< 0.01), we completed univariate meta-regression using the following independent variables: document date, type of document, country, sample size, attrition, study design, maternal age, marital status, education, employment, income, substance abuse history, previous substance abuse treatment, mental and physical health, involvement with the legal system, child age, custody, involvement with child protection services, positive toxicology at birth, and treatment program characteristics (e.g., program for pregnant and/or parenting women, planned length of treatment, intensity of treatment, residential or outpatient, type of services). These variables did not significantly moderate the substance use effect. It is important to note that, due to missing data and our inability to include all studies in all analyses, these analyses may have been underpowered.

### Studies comparing integrated programs to non-integrated programs

There were 10 studies comparing substance use for women participating in integrated and non-integrated programs. As can be seen in Table 3, 9 out of 16 measures indicated better outcomes for integrated programs and most of these effect sizes were small and non-significant. We combined studies with the most common measures of maternal substance use (urine toxicology and self-report abstinence, i.e., percent not using). Four studies examining urine toxicology indicated no significant differences between integrated and non-integrated programs. Carroll et al. [39] found that 71% of integrated and 76% of non-integrated program clients had negative urine screens (n = 7 in each group). Similarly, Barkauskas, Low, & Pimlott [44] found that 95% of integrated and 97% of non-integrated program clients had negative urine screens (n = 37 and 35, respectively). Chang, Carroll, Behr, & Kosten [45] examined 6 integrated and 6 non-integrated program clients and found that more integrated program clients had negative urine screens (41% and 24%, respectively). Luthar et al. [41] compared a relational psychotherapy mothers group plus standard methadone treatment (treatment group) with a recovery training plus standard methadone treatment (control group) on opiate and cocaine screens (n = 60 and 67, respectively). No significant group differences were found on opiate or cocaine screens. Taken together, the combined effect size data for these 4 studies suggest that the percentage of clients with negative urine screens in integrated and non-integrated programs was not significantly different (*d *= -0.09, *CI *= -0.412 to 0.224, *z= *-0.58, *p *= 0.56). Cochran's chi square test indicated that there was no statistically significant heterogeneity among studies, *Q *(3) = 0.66, *p *= 0.88.

There were two studies comparing self-reported abstinence for women in integrated and non-integrated programs. Sowers, Ellis, Washington, & Currant [48] examined differences in abstinence for integrated residential treatment and non-integrated day treatment. A moderate effect was found (*d *= 0.33) but was not statistically significant. Suchman, Mayes, Conti, Slade, & Rounsaville [49] found a small, non-significant effect (*d *= 0.15) when comparing abstinence for women in women-only outpatient treatment programs with or without parenting services. Taken together, the combined effect size data suggest that the percentage of clients reporting abstinence in integrated and non-integrated programs was not significantly different (*d *= 0.22, *CI *= -0.231 to 0.672, *z= *0.96, *p *= 0.34). There was no statistically significant heterogeneity among studies, *Q *(1) = 0.158, *p *= 0.691.

## Discussion

This systematic review and meta-analysis addressed the effectiveness of integrated programs for women with substance use issues and their children in improving maternal substance use outcomes. In the two studies of women in integrated programs versus no treatment, effect sizes for substance use (urine toxicology and percent using drugs or alcohol) significantly favored integrated programs and ranged from 0.18 to 1.41, which are small to large in strength. In the five cohort studies involving measures of severity of drug and alcohol use for women in integrated programs, the average effect sizes were 0.64 and 0.40, respectively. In the four cohort studies of number of days of substance use for women in integrated programs, the average effect size was 0.52. These cohort study effects were statistically significant, medium size, and indicated that integrated programs are effective in reducing the severity of substance use and the number of days of substance use from beginning to end of treatment. These findings are consistent with research that has shown that substance use treatment programs are generally effective in reducing substance use [59-61].

In our meta-analysis of studies comparing women who participated in integrated programs to women who participated in non-integrated programs, there were four studies assessing urine toxicology and two studies assessing self-reported abstinence. Overall effect sizes were -0.09 and 0.22 and both were nonsignificant. These results are similar to Orwin et al.'s [18] meta-analysis of studies comparing women-only programs to mixed gender programs, in which substance use effects favoring women-only programs were small and non-significant. The lack of significant differences between integrated and non-integrated programs may, in part, reflect methodological limitations, including issues relating to measurement of substance use.

### Operationalization of substance use

The most common measures of substance use in treatment studies are abstinence measures (e.g., urine toxicology, percent using) or frequency of use (e.g., number of days of use). While these measures provide information about substance use, they do not reflect the complexity of substance use and may not fully reflect changes made by women in treatment. For example, frequency measures do not account for changes in quantity (e.g., the number or strength of drinks or level of intoxication) or type of substance used. Similarly, urine toxicology measures are useful for measuring abstinence as reflected by recent substance use (past 2-3 days), but cannot provide information about reduction in use or changes in the pattern of use over a longer time period [38]. Reduced use would have particular significance, for example, if it was associated with reduced impairment or reduced use of illegal substances [48]. Therefore, substance use is best represented as a pattern of behavior reflecting variables such as quantity, frequency, duration of use, impact, and type of substance [62]. In our meta-analysis, studies involving a multi-dimensional measure of substance use (the Addiction Severity Index) had significant, medium-sized effects whereas studies involving unidimensional measures of abstinence (urine toxicology) had small, nonsignificant effects. Therefore, the manner in which substance use is operationalized and measured may impact on the size of observed effects.

### Theoretical specificity of outcome measures

The extent to which substance use measures are theoretically specified to the treatment model also may impact effects. For example, programs using a harm-reduction approach to treatment that only use measures of abstinence to assess change may potentially miss clinically significant improvements in substance use. Urine toxicology is the most commonly used biological assay method for illicit drugs. Urine toxicology allows one to assess what percentage of participants have not been using drugs in the immediate past. However, at least some of "abstinent" participants may have used drugs over the total assessment period [38]. Abstinence-based measures also cannot account for reduced substance use or changes in substances used. Therefore, it is possible that abstinence measures may over- or underestimate substance use. The National Institute on Alcohol Abuse and Alcoholism, Project MATCH Research Group [63] found that while participants decreased their alcohol consumption, most continued to use alcohol at a decreased level at one year follow up. Despite the advantages of urine toxicology as an objective measure of abstinence, substance use treatment studies, particularly those adopting a harm reduction treatment model, should include multi-dimensional measures of substance use to fully capture the changes made by women.

### Reliability of self-report measures

Self-report measures are commonly used in the field. The reliability of self-report measures of substance use over a specific time period since leaving treatment (for example, the past 6 months) is open to question. While underreporting is common [64,65], there is some evidence that treatment participants may be more likely to report that they have used drugs than those who have not been in treatment [38,66]. These reporting biases may obscure differences between groups and impact observed effects. It is also possible that self-report measures of substance use may be less reliable and valid than self-reports of other outcomes [67,68]. In part, this may explain why Orwin et al. [18] found larger effects for maternal and child well-being outcomes than substance use in their meta-analysis comparing women-only to mixed gender treatment programs.

### Limitations

There were a number of challenges encountered in conducting this meta-analysis, including few comparison group studies, low levels of study quality, and a high level of missing data.

### Few comparison group studies

The majority of studies included in the meta-analysis involved a cohort research design, with relatively few studies examining differences between integrated and non-integrated programs. While we were able to include data comparing 3111 women in our review, the size of the observed effects may have been impacted by the small number of studies. As with the substance abuse treatment field generally, most program evaluations involved non-random designs and tested correlational rather than causal relations [38]. Finally, the small number of studies made it difficult to explore moderators of treatment effect and to determine what treatment is best for whom under what circumstances.

### Study quality

Studies included in the meta-analyses were assessed as being of low to moderate quality, although it was unclear if the scores reflected study quality per se or the reporting of study quality elements. It is possible that the study quality ratings, particularly for the randomized trials, may have been underestimated. The Jadad scale used to assess the quality of randomized trials is a very conservative measure of study quality that addresses methodological characteristics such as studies being double blind. Such characteristics may be impractical to implement in substance abuse treatment research. Despite this limitation, there are areas of study quality that can be improved. For example, only 48% of studies included information about attrition. The manner in which attrition is addressed statistically (e.g., omitting these participants, intent to treat method) has the potential to limit the validity of results [69]. An emphasis on high quality randomized or quasi-experimental designs comparing clearly defined integrated and non-integrated treatments is needed to move the field forward.

### Missing study information

It was surprising how often essential information about a study or program was unavailable. Missing study information needed to calculate effect sizes led to some studies not being included in the present meta-analysis. It also impeded our exploration of participant and program characteristics that might moderate substance use outcomes. Ensuring the availability of essential information to describe studies for future meta-analyses on integrated programs could be accomplished by improvements in the editorial review process and creation of a registry of funded studies that would require submission of standard information (such as the Cochrane Collaboration on health care intervention) [38].

## Conclusion

The findings from this meta-analysis suggest that integrated programs for women with substance use issues and their children are associated with significant reduction in substance use.

However, integrated programs were not associated with significantly more reduction in substance use compared to non-integrated programs. While these findings suggest that the current evidence base does not support integrated over non-integrated programs for reduction of substance use, there are a number of important limitations raised by this meta-analysis and synthesis that merit attention from a policy perspective. Given the few comparison group studies and low levels of study quality seen in the current review, scarce research funding resources need to be directed towards high quality prospective studies with randomized designs and larger samples. The field will advance only as researchers conduct high quality studies that manipulate treatment conditions, rather than examining them post hoc, and that take into account the diversity of substance-using populations. Reporting practices also need to be improved and standardized to include full descriptions of the target population and the intervention program.

To our knowledge, this meta-analysis is the first systematic quantitative review of studies evaluating the specific impact of integrated treatment programs on maternal substance use. Given that approximately one third of people with drug dependence are women of child-bearing age [10], substance use during pregnancy is a major public health concern [1] and burden of suffering due to maternal substance abuse is great, the findings from this study are noteworthy and support funding for further research on integrated programs for women with substance abuse issues and their children.

## Competing interests

The authors declare that they have no competing interests.

## Authors' contributions

KM., AN, WS, LT, JH, and AS contributed to the development of the meta-analysis. KM and AN developed the eligibility criteria and codebook, with input from all other authors. AS completed the literature search. KM, AS, and JL participated in the coding of articles. Study quality measurement was completed by AN, LT, JL, and AS. KM and AS conducted the statistical analyses, with consultation from LT and AN. KM and AN wrote the first draft of the manuscript. All authors contributed to and have approved the final manuscript.
